# The Further You Are, the Closer You Get: Environmental Similarity Connects Aquatic Invertebrate Communities Across the Terrestrial Landscape in a Large River Network

**DOI:** 10.1002/ece3.74302

**Published:** 2026-09-21

**Authors:** Jared W. Freedman, Theodore A. Kennedy, David A. Lytle

**Affiliations:** ^1^ Department of Integrative Biology Oregon State University Corvallis Oregon USA; ^2^ U.S. Geological Survey Southwest Biological Science Center, Grand Canyon Monitoring and Research Center Flagstaff Arizona USA

## Abstract

Biodiversity is structured on the landscape through a combination of spatial, environmental, and stochastic factors. In freshwater river networks, the interaction of the linear river network and the three‐dimensional terrestrial landscape creates multiple pathways for ecological connectivity that affect aquatic community structure through habitat filtering and dispersal dynamics. Here, we use the high throughput capacity of environmental DNA (eDNA) metabarcoding to explore the influence of spatial relationships and environmental heterogeneity across an expansive and diverse landscape. We collected invertebrate eDNA samples from 70 aquatic habitats in the lower Colorado River Basin (LCRB), ranging from small, free‐flowing headwater tributaries to anthropogenically‐altered high order rivers, spanning five major drainage regions. Communities displayed high alpha and beta diversity among the drainage regions, structured by a combination of spatial, environmental, and stochastic factors. A novel distance‐decay framework adapted from population genetics revealed the influence of terrestrial pathways of ecological connectivity among communities throughout the network. Hierarchical clustering identified patterns of convergent similarity in headwater tributaries despite extreme river network distances separating sites. These results reveal the dual influence of spatial and environmental metacommunity dynamics in aquatic invertebrate communities in the LCRB and the specific influence of interbasin terrestrial connectivity across large spatial scales. Overall, this highlights how terrestrial landscapes can shape convergent patterns of biodiversity in river networks.

## Graphical abstract

Ecological connectivity in freshwater ecosystems can be structured by river network, the terrestrial geography, and environmental conditions. In the Lower Colorado River Basin, aquatic invertebrate communities are influenced by all three dynamics, and environmental heterogeneity is the strongest observed driver of community composition.

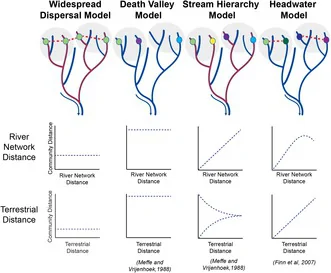

## 1 Introduction

Ecological connectivity—the ecologically relevant movement of organisms across the physical structure of the landscape (Taylor et al. 2006)—is a major determinant of biodiversity. Connectivity influences many aspects of community assembly, including dispersal, environmental habitat filtering, and biotic interactions (Krosby et al. 2010; Leibold et al. 2004; Suzuki and Economo 2021), forming the foundation of biogeography and metacommunity ecology (Leibold and Chase 2018; Nekola and White 1999; Whittaker 1960). A central pattern in both disciplines is the generally positive relationship between the rate of change in biological communities and environmental and spatial landscape gradients (i.e., distance decay; Nekola and White 1999, Soininen et al. 2007). Different modes of ecological connectivity contribute to the biotic and abiotic processes that drive this pattern (Tonkin et al. 2018). Spatial landscape gradients, such as Euclidean geographic distances, watercourse distances, or topographically weighted distances, are frequently used as proxies for dispersal (Heino, Alahuhta, et al. 2017), while gradients in environmental conditions are often related to differences in ecological niches (Nekola and White 1999). As such, analysis of these landscape metrics can provide insight into the ways in which ecological connectivity shapes the structure of biological communities.

In riverine ecosystems, ecological similarity among aquatic communities can be structured by hydrological connectivity along the river network, by terrestrial connectivity across overland dispersal pathways, by nonspatial environmental similarity, or by a combination of these landscape characteristics (Cañedo‐Argüelles et al. 2015; Heino, Melo, Siqueira, et al. 2015; Tonkin et al. 2018). The branching topology and flow directionality of rivers connect aquatic habitats as points in a dendritic network, particularly when organisms are constrained to the aquatic realm or rely on riparian corridors for dispersal (Benda et al. 2004). Aquatic habitats also exist as points on the terrestrial landscape where the geographic relationships among habitats differentially affect organisms with aerial, terrestrial, or mediated dispersal (Peterson et al. 2013; Tonkin et al. 2018). Meanwhile, environmental characteristics are influenced by both the river network and the terrestrial landscape, creating dynamic interactions between the environment and the aquatic landscape (Heino, Melo, and Bini 2015). For example, habitats at the extremities of river networks, such as small headwater streams, may be found in geographically proximal and environmentally similar locations in the terrestrial landscape (Jones and Schmidt 2017; Meyer et al. 2007). On the other hand, high‐order mainstem rivers can exhibit similar environmental conditions even across large spatial distances (Benda et al. 2004; Jones and Schmidt 2017).

The multiple modes of habitat connectivity in river networks influence the structure of aquatic communities, particularly when landscape structure limits connectivity along one or more pathways (Cañedo‐Argüelles et al. 2015; Heino, Soininen, et al. 2017). The relative importance of different types of connectivity influences the metacommunity dynamics acting throughout a river network (Henriques‐Silva et al. 2019; Schmera et al. 2018). Species sorting, a local‐scale metacommunity archetype in which community assembly is dictated by tolerances to local environmental conditions, is expected to be highly influential in headwater and other small rivers and streams (Brown and Swan 2010; Heino, Soininen, et al. 2017; Heino, Melo, Siqueira, et al. 2015). Communities in these habitats experience high isolation and inhabit unique environments that are strongly influenced by terrestrial landscape topography (Clarke et al. 2008; Fagan 2002; Miller et al. 2015; Ward 1994). On the other hand, communities in higher‐order rivers are expected to reflect regional‐scale metacommunity archetypes of dispersal limitation and mass effects (Brown and Swan 2010; Tonkin et al. 2018). Communities in higher‐order rivers are more centrally located in the river network, leading to greater river network connectivity (Brown and Swan 2010; Campbell Grant et al. 2007). The location of a habitat within the river network therefore plays a major role in structuring these metacommunity dynamics (Brown and Swan 2010; Schmera et al. 2018), although the importance of network position is complicated by a number of factors, including overland dispersal among headwater drainage basins (Göthe et al. 2017; Schmera et al. 2018) and local variation in environmental conditions (Heino, Soininen, et al. 2017).

**FIGURE 1:**
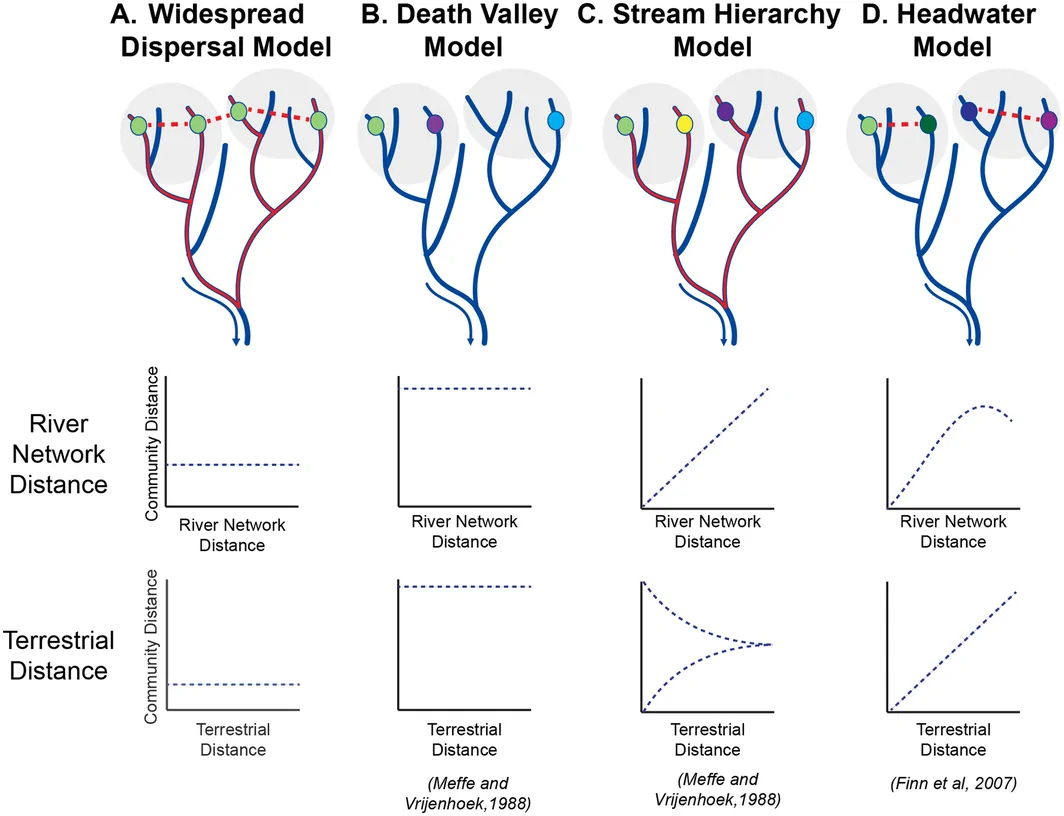
Conceptual distance‐decay framework of ecological connectivity between aquatic habitats, derived from models of population genetic connectivity. River networks in the top row show hypothesized connectivity pathways for each model, denoted with red lines. Circles show communities, with similar hues representing similar community structures. (A) Widespread Dispersal Model: When dispersal ability is sufficient to reach all habitats within the region, community dissimilarity will be low across all measures of spatial distance. (B) Death Valley Model: When dispersal ability is insufficient to reach even nearby habitats, community structure will be subject to stochastic drift, and dissimilarity will be high at all geographic distances. (C) Stream Hierarchy Model: When dispersal relies on the river network for connectivity, there will be a positive relationship between community dissimilarity and river network distance and a biphasic convergence with Euclidean geographic distance due to the close geographic proximity of hydrologically distant headwater sites. (D) Headwater Model: When dispersal occurs across terrestrial pathways, there will be a positive relationship between community dissimilarity and Euclidean geographic distance, while river network distance will display a linear relationship at short to moderate distance and begin to decrease at large network distances due to the geographic proximity of hydrologically distant headwater habitats.

To account for the complexity and heterogeneity of riverine landscapes, conceptual models of ecological connectivity in river networks can provide a descriptive framework to understand how different models of connectivity will affect community assembly. A suite of models developed in the population genetics literature provide a comprehensive, although not exhaustive, description of the potential connectivity pathways in dendritic river networks (Figure 1; Hughes et al. 2009, Tonkin et al. 2018). Although these models were developed for population genetic analyses of individual populations, the processes that govern population genetic structure (selection, mutation, gene flow, and genetic drift) closely resemble the metacommunity dynamics that govern community structure (Tonkin et al. 2018; Vellend 2010, 2005), enabling the application of these models to understand the role of connectivity in the assembly of aquatic communities. The widespread dispersal model (WDM; Hughes et al. 2009) predicts high connectivity across aquatic and terrestrial pathways, allowing mass effects to reduce community across all landscape distances (Figure 1A). Conversely, the high isolation Death Valley model (DVM; Meffe and Vrijenhoek 1988) predicts low connectivity across aquatic and terrestrial pathways will cause high community dissimilarity across all landscape distances due to environmental heterogeneity and neutral stochasticity (Figure 1B). The stream hierarchy model (SHM; Meffe and Vrijenhoek 1988) predicts that communities are structured by river network connectivity, with regional metacommunity archetypes such as mass effects and dispersal limitations dominating (Figure 1C). Finally, the headwater model (HWM; Finn et al. 2007) predicts that communities are structured by terrestrial connectivity and will therefore display a positive linear relationship with terrestrial distance and a hump‐shaped relationship with aquatic connectivity (Figure 1D). Due to the dynamic interactions of hydrological, terrestrial, and environmental connectivity, these models are not mutually exclusive (Tonkin et al. 2018). Rather, they represent archetypal patterns of community structure that can guide interpretation of patterns in aquatic community structure. Spatially explicit analyses of community structure, such as distance‐decay relationships (e.g., Cañedo‐Argüelles et al. 2015) and variance partitioning analysis (e.g., Schmera et al. 2018), can be used to describe community diversity patterns associated with these connectivity models and thereby infer potential metacommunity dynamics influencing aquatic communities (Figure 1).

Rivers in desert ecosystems exhibit particularly harsh contrast between aquatic and terrestrial landscapes, leading to ecological disjunctions between hydrologically connected habitats and increased likelihood of stochastic disturbances such as floods and droughts (Bogan and Lytle 2011; Jones and Schmidt 2017). Furthermore, rivers in arid landscapes are disproportionately affected by anthropogenic habitat alterations such as dam impoundments and diversions (Nilsson et al. 2005), which alter biodiversity and artificially constrict aquatic communities (Abernethy et al. 2021; Kennedy et al. 2016; Minckley 1991). The Colorado River is one of the largest desert river networks in the world, draining over 640,000 km^2^ of the southwestern United States.

The ability to make inferences regarding landscape‐scale patterns of community structure is predicated on the sampling of many communities in diverse habitats. To do so, we used environmental DNA (eDNA) metabarcoding of aquatic invertebrates, allowing for quantification of community structure from the genetic material obtained from water samples (Bohmann et al. 2014; Lodge et al. 2012). eDNA allows for standardized sampling of invertebrate communities across a diverse array of habitat types, enabling widespread spatial coverage with a single methodological approach that could not otherwise be obtained by a single conventional sampling method (Altermatt et al. 2020). Here, we integrate a spatially explicit distance‐decay framework with existing models of landscape genetic connectivity to investigate whether aquatic invertebrate community structure across the lower Colorado River Basin (LCRB) is influenced by spatial relationships (either aquatic or terrestrial) among communities or by heterogeneous local habitat conditions found in each habitat. We also test for biogeographic patterns of similarity across the LCRB using a hierarchical clustering approach.

## 2 Methods

### 2.1 Conceptual Framework

To identify the dominant modes of ecological connectivity in dendritic river networks, we adapted a novel implementation of descriptive connectivity models initially developed for population genetic relationships by Meffe and Vrijenhoek (1988) and Finn et al. (2007), and proposed for analysis of metacommunity structure in a review by Tonkin et al. (2018). Due to the variety in functional forms of distance decay relationships (DDR; Nekola and McGill 2014), our framework focuses on general trends in the DDR slope, allowing its application to various river networks and taxonomic groups. The WDM predicts low dissimilarity across all landscape distances due to high dispersal across both aquatic and terrestrial pathways (Figure 1A). Conversely, the high isolation DVM predicts high community dissimilarity across all landscape distances due to isolation and environmental heterogeneity (Figure 1B). The SHM predicts that communities are structured by river network connectivity and will therefore display a positive relationship with river network distance and a biphasic converging relationship with terrestrial distance (Figure 1C). The predicted biphasic relationship with terrestrial distance is produced by headwater sites with terrestrial proximity possessing either short watercourse distances (within‐basin; low dissimilarity) or long watercourse distances (between‐basin; high dissimilarity). The HWM predicts that communities are structured by terrestrial connectivity and will therefore display a positive relationship with terrestrial distance and a hump‐shaped relationship with aquatic distance (Figure 1D). The predicted hump‐shaped relationship with aquatic distances is produced by the convergence of headwater habitats in terrestrial space across drainage basins, where sites at the extremity of watercourse distances will be found in similar headwater landscapes.

### 2.2 Study Area

The lower Colorado River Basin, defined as the drainage of the Colorado River below Glen Canyon Dam by the 1922 Colorado River Compact, drains a desert and semi‐arid landscape with several ecoregions, including the Mojave and Sonoran Deserts and semi‐arid forests and scrubland of the Colorado Plateau (Cañón et al. 2011). Freshwater habitats in this ecosystem exhibit diverse environmental characteristics, ranging from small first‐order headwater tributaries to large seventh‐order rivers. Precipitation across the LCRB averages 32 cm per year, although there is high temporal and spatial variation in precipitation across the basin (National Oceanic and Atmospheric Administration National Centers for Environmental Information, retrieved June 2026). Many low‐order tributaries, as well as some larger rivers like the Colorado River within Grand Canyon, are bound by steep canyon topography, creating opportunities for ecological isolation among subdrainages (Bogan and Boersma 2012; Cañedo‐Argüelles et al. 2015). Furthermore, major anthropogenic alterations to the river network alter the natural flow regime throughout the lower Colorado River Basin, with a series of six major hydroelectric and diversion dams along the Colorado River and many other dams and diversions of various sizes in other parts of the basin. These freshwater habitats range from protected wilderness areas to heavily modified agricultural land and densely populated urban areas, and are affected by numerous anthropogenic alterations (dams, diversions, agricultural). Headwater habitats possess diverse communities of aquatic invertebrates (Hockett 2023; Oberlin et al. 1999), while higher‐order rivers like the Colorado, Salt, and Gila rivers support lower diversity and are affected by substantial anthropogenetic influence (Abernethy et al. 2021; Kennedy et al. 2016). The rivers and streams of the LCRB present an ideal system for studying how aquatic invertebrate communities are shaped by local environmental characteristics and regional spatial factors.

We collected eDNA samples from 70 sites across the lower Colorado River Basin from March to May 2022, ranging from 1st order tributary streams to the 7th order Colorado River (Figure S1). Samples were taken in collaboration with two major research projects sponsored by the U.S. Forest Service and the U.S. Geological Survey, spanning five major geographic regions: the mainstem of the Colorado River, Grand Canyon tributary streams, the Verde River, Mogollon Rim headwater streams, and the Salt and Gila Rivers (Table S1). Sites within the Grand Canyon ecosystem were sampled at boat‐accessible locations in April 2022. Sites within the Verde River basin, the Gila and Salt Rivers, and the lower Colorado River segments below Hoover Dam were sampled at vehicle‐accessible locations during March and April 2022.

### 2.3 Sample Collection

At each sampling location, four replicate water samples were taken from the surface of aquatic habitats with positive down‐channel flow. For each sampling replicate, 1 L of stream water was collected in sterile 1.5 L roll‐top bags (Whirl‐Pak, Austin, TX, USA) while wearing nitrile gloves, and sampling locations were approached from downstream to prevent contamination between samples. Water samples were either processed on site or stored in a cooler until filtration later in the day (< 8 h). eDNA was obtained from water samples by filtering onto a 0.45 μm MCE filter (Sterlitech, Auburn, WA, USA) using a vacuum pump manifold. Filters were then preserved in 1.5 mL of ATL buffer (QIAGEN, Germantown, MD, USA) and stored in an insulated container at room temperature until extraction.

### 2.4 DNA Extraction, Sequencing, and Bioinformatics

eDNA extractions were performed using a modified protocol for the DNeasy Blood and Tissue kit (QIAGEN; Appendix: Methods S1). To increase DNA yield in each sample, we combined two filters from each site into one extracted sample, resulting in two 2‐L sample replicates of extracted eDNA per site. Extracted DNA was then purified of polymerase chain reaction (PCR) inhibitors using the One‐Step PCR Inhibitor Removal Kit (Zymo Research, Irvine, CA, USA).

We used a two‐stage PCR approach for library preparation of cytochrome oxidase I (COI) amplicons, and the PCR amplification and library preparation protocols are fully described in the Appendix (Methods S2). Three PCR replicates were performed for each of two sample replicates, generating six total replicates per sample location. In the first stage, we used the fwhF2/EPTDr2n primer set to amplify a 142 bp fragment of the invertebrate COI gene (Leese et al. 2021). In the second stage, we used fusion primers to add unique dual indices and the Illumina adapter tails before pooling these sub‐libraries in equimolar amounts. eDNA libraries were sequenced by Oregon State University's Center for Quantitative Life Sciences (CQLS) using the Illumina NextSeq 2000 150 bp pair end P1 kit.

Sequencing reads were demultiplexed using Illumina software, and the JAMP v0.77 metabarcoding pipeline was used to trim, filter, and cluster reads into operational taxonomic units (OTUs) with < 3% sequence similarity (Elbrecht et al. 2018). OTUs identified in only a single replicate were removed from the dataset. For full metabarcoding pipeline, refer to the Appendix (Methods S3). OTUs were assigned taxonomic identification by aligning OTU sequences to the BOLD database using BOLDigger v2.0.4 (Buchner and Leese 2020). Identified OTUs with a reference similarity threshold < 85% were excluded from further analysis. Several field replicates produced low reads for all three PCR replicates, and these samples were removed from further analysis (VRBC_1, VRBC_2, VRBH_1, VRWC_2). The resulting community dataset was transformed to presence‐absence.

### 2.5 Statistical Analysis

All statistical analyses were carried out in R statistical software (version 4.5.1; R Core Team 2025). We used hierarchical clustering analysis of pairwise Sorensen dissimilarity between communities to distinguish distinct units of community similarity. Hopkins statistic tests revealed our data to be highly suitable for clustering (mean Hopkins statistic after 9999 iterations = 0.878). We calculated hierarchical dendrograms from presence‐absence community data and validated the optimal number of clusters by calculating Internal and Stability measures: connectivity, Dunn index, silhouette coefficient, average distance between means, and average proportion of non‐overlap. Optimal cluster configurations were projected on a map of the LCRB displaying sampling locations, rivers, and the topographic structure of the surrounding landscape.

Distance decay of community Sorensen dissimilarity was compared to pairwise distances calculated for four habitat characteristics: Euclidean geographic distance, topographic least‐cost distance, river network distance, and environmental distance. Sorensen dissimilarity was calculated from presence‐absence community data using the *vegdist* function of R package *vegan* (Oksanen et al. 2026). Five significant environmental variables (drainage area, annual precipitation, elevation, stream type, and 100‐year flood magnitude) were identified using forward selection of model parameters with the *ordistep* function of the R package *vegan* (Oksanen et al. 2026). These variables were submitted to principal component analysis (PCA), and the first five axes were retained (explained variance = 100%), and composite environmental distances between sites were calculated as the Euclidean distance between their respective PCA scores (Figure S2). Pairwise Euclidean geographic distances were calculated from straight‐line distances between site coordinates. Pairwise topographic least‐cost distances were calculated using a slope‐derived cost surface from a digital elevation model (DEM) with the R package *gdistance* (Van Etten 2017). Pairwise river network distances were calculated using the “OD Matrix from Layers as Table (m:n)” tool in the QGIS package QNEAT3 (Raffler 2018). Relationships between community dissimilarity and pairwise habitat distances were assessed using generalized dissimilarity models (GDMs) that allow for nonlinear relationships (Ferrier et al. 2007), using the R package *gdm* (Fitzpatrick et al. 2025). Four univariate GDMs were fit for each distance metric independently of the community dissimilarity matrix, and the significance of each relationship was determined using a permutation test with 9999 permutations, with the resulting *p*‐value being the proportion of permutations where randomized predictor data performed better than actual predictor data. A multivariate full model comparing community dissimilarity with environmental distance, topographic least‐cost distance, and river network distance was calculated as well, and significance was calculated using a permutation test with 9999 permutations. Topographic distance was used instead of Euclidean distance in the multivariate model due to the strong correlation between the two metrics (Spearman rank correlation = 0.958), and the increased landscape information contained in topographic distance.

The shape and fit of these distance decay relationships were qualitatively assigned to one of the four models of ecological connectivity (Finn et al. 2007; Meffe and Vrijenhoek 1988). Scatterplots of distance decay relationships for all four distance metrics were plotted and fitted with a nonparametric locally estimated scatterplot smoothing (LOESS) regression curve. This allows for identification of distance decay trends regardless of the functional form of the distance decay relationship, which can vary among taxonomic groups and spatial scales (Nekola and McGill 2014). Models were characterized based on the framework outlined in *Conceptual framework* and Figure 1.

The relative contributions of geographic space, river network connectivity, and local environmental characteristics to aquatic invertebrate community composition were calculated using multi‐matrix distance‐based redundancy analysis (dbRDA) with hierarchical partitioning, as implemented in the R package *rdacca.hp* (Lai et al. 2022). Moran's eigenvector maps (MEMs; Dray et al. 2006) were used to model spatial structures between sampling sites and were calculated separately for Euclidean geographic space (Figure S3) and river network space (Figure S4) using *scores.listw* in the *adespatial* R package (Dray et al. 2026). MEMs that significantly explained variation in community composition were identified using *forward.sel* (9999 permutations). Due to the large spatial extent of the LCRB and the multiple modes of dispersal utilized by aquatic invertebrates, symmetrical MEMs were calculated for the river network. The significant MEMs, along with the five environmental variables identified by forward selection, were used as predictor matrices for dbRDA on Sorensen dissimilarity. Hierarchical partitioning of predictors (*permu.hp* with 9999 permutations) was used to calculate the relative contribution of each predictor across all possible model subsets, including individual and joint fractions, thereby estimating the independent importance of each habitat characteristic.

## 3 Results

### 3.1 Alpha Diversity

**FIGURE 2:**
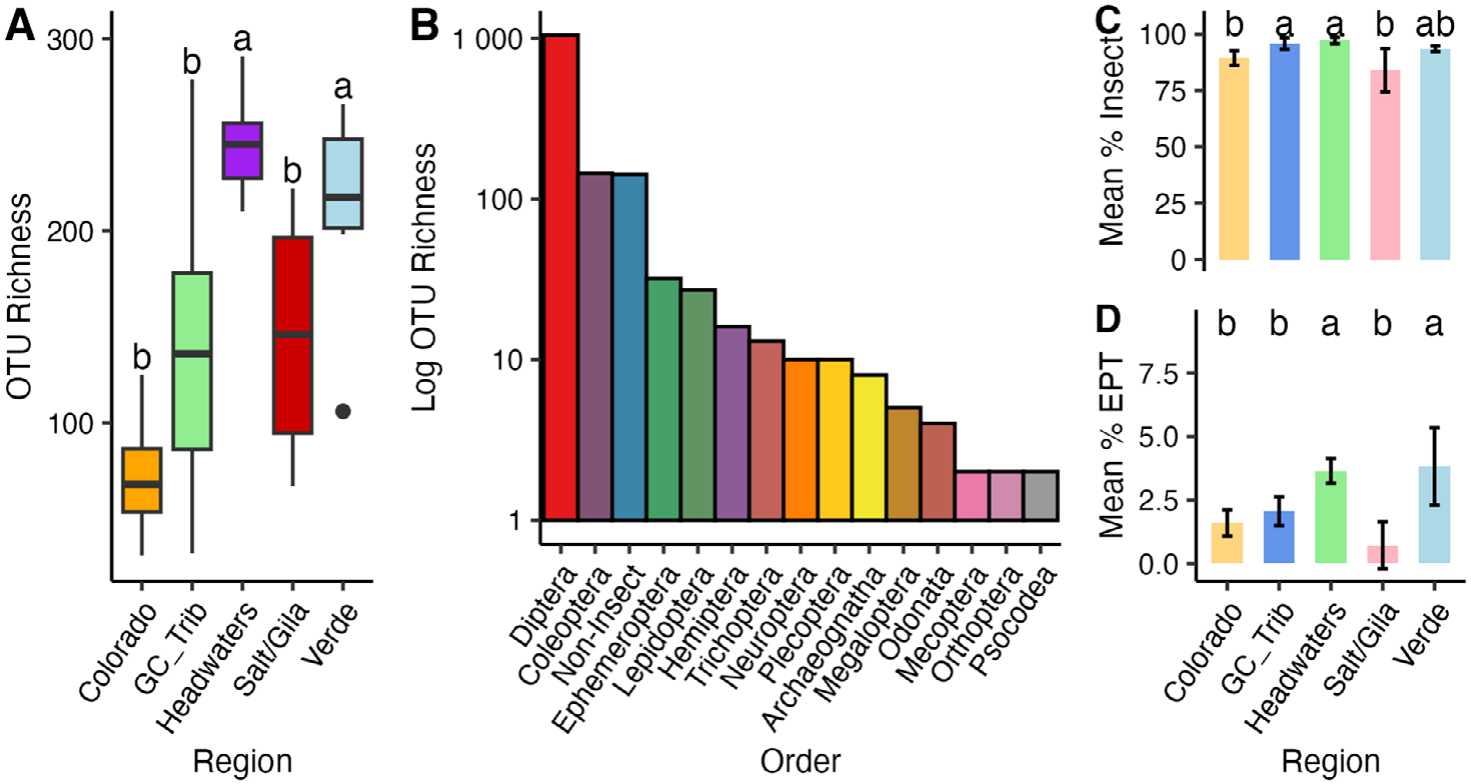
Alpha diversity of lower Colorado River Basin communities. (A) Mean operational taxonomic unit (OTU) richness of sampled aquatic habitats in each of five predefined drainage regions. OTU richness differed significantly between drainage regions. Differences in means were compared with Tukey's range test, and significant differences are indicated by letters above each box. Boxes correspond with upper and lower quartiles, the bisecting line corresponds to median, and the whiskers correspond to 1.5 interquartile range. (B) Order‐level identification of observed OTUs. *Y*‐axis shows log‐scaled OTU richness. For simplicity, all OTUs identified to a class other than Insecta were given the order‐level designation “Non‐Insect.” (C) Percent of OTUs identified to the class Insecta. Most identified OTUs belonged to Insecta, although percentages differed significantly by drainage region. (D) Percent of OTUs identified to the orders Ephemeroptera, Plecoptera, and Trichoptera (EPT). Proportion of EPT taxa differed significantly by drainage region and were highest in the Verde River and its Mogollon Rim tributaries. Error bars for (C) and (D) represent the 95% CI for means within each drainage region.

Following sequence filtering and denoising, we retained an average of 284,635 reads per replicate. Across all sites sampled, we identified 1445 aquatic‐associated invertebrate OTUs with reference similarity > 85%. Alignment of OTU sequences with the BOLD reference database identified 335 species‐level classifications, 311 genus‐level classifications, and 655 family‐level classifications. Mean OTU richness differed significantly between predefined drainage regions (ANOVA, *F* = 27.67, *p* < 0.0001; Figure 2A), with the Verde River and Mogollon Rim headwater communities exhibiting significantly higher richness and the Colorado River exhibiting significantly lower richness. Drainage regions also differed significantly in the proportion of taxa belonging to the class Insecta (ANOVA *F* = 5.21, *p* = 0.0011; Figure 2C) and to the insect orders Ephemeroptera, Plecoptera, and Trichoptera (ANOVA *F* = 8.08, *p* < 0.0001; Figure 2D). Diptera were by far the most common order identified (Figure 2B), with 72% of all OTUs identified in the LCRB belonging to Diptera (*n* = 1042).

### 3.2 Hierarchical Clustering

**FIGURE 3:**
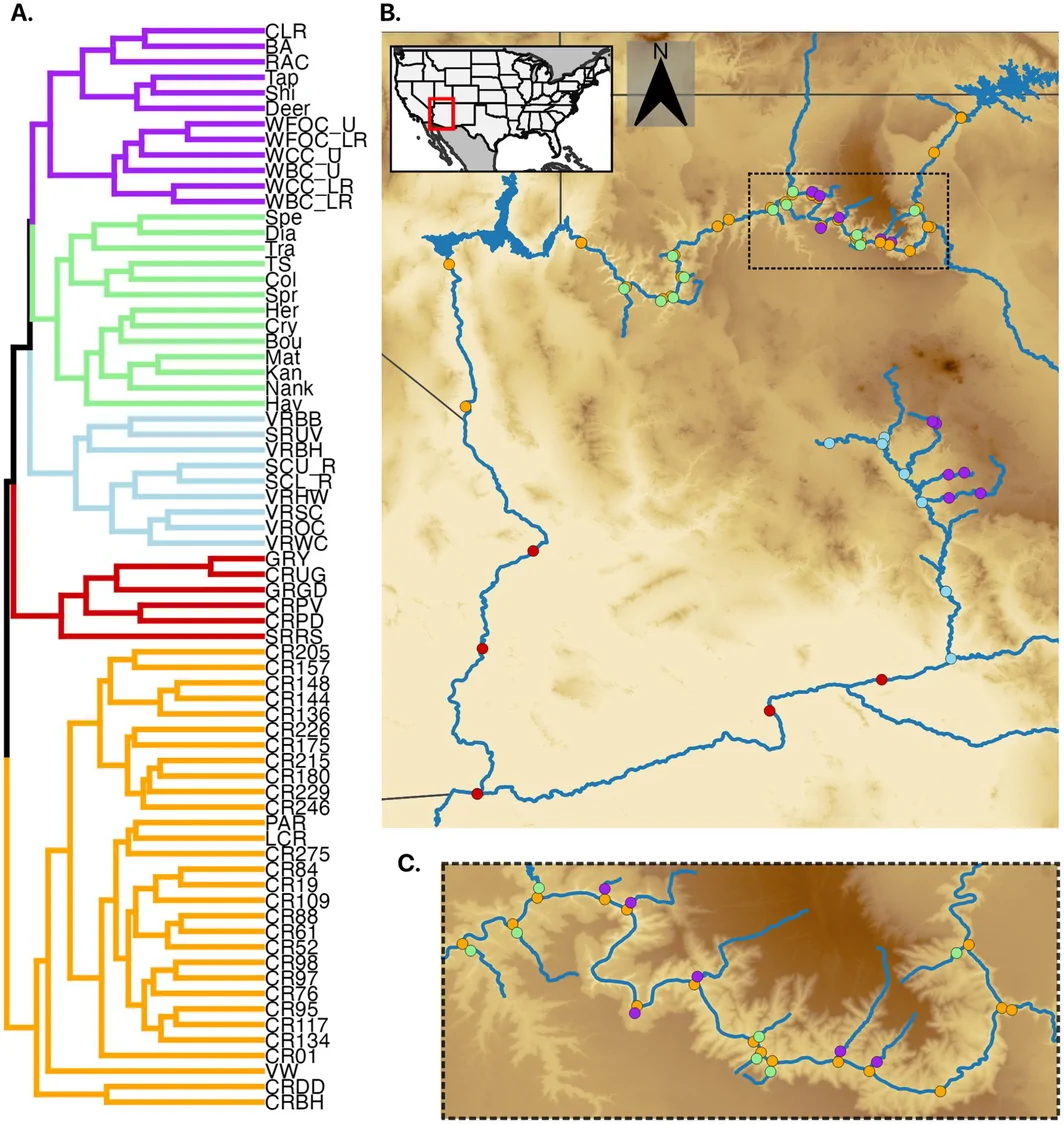
Five group hierarchical clustering of aquatic invertebrate communities in the lower Colorado River Basin (LCRB). A five‐cluster solution supported by one validation method: Dunn Index. (A) Dendrogram of community similarity displaying five clusters. (B) Map of sampled communities in LCRB, with point colors corresponding to cluster identity. (C) Map of sites in Grand Canyon, highlighting the area inside the dashed box on the larger map. Five clusters generally follow a priori regional basin designations, with the major exceptions of a cluster consisting of lower Colorado River and Salt/Gila communities (red), and a cluster consisting of tributary habitats in the Mogollon highland and a subset of Grand Canyon tributaries. Base map is from USGS Global Multi‐resolution Terrain Elevation Data 2010 (GMTED2010), and river shapefiles are from USGS National Hydrography Dataset 2023.

Hierarchical clustering of communities based on compositional similarity identified both two‐ and five‐cluster solutions for defining biogeographical units of similarity in the LCRB. The two‐cluster solution (supported by connectivity, silhouette coefficient, average distance between means, and average proportion of non‐overlap) produced a clear delineation of Colorado River communities from the remainder of the communities (Figure S5). The five‐cluster solution (supported by Dunn Index) further delineated the large non‐Colorado River cluster into four smaller groups. These smaller clusters generally align with a priori regional basin designations with a few exceptions (Figure 3). Communities in the lower Colorado River (below Parker Dam, below Palo Verde Dam, and above the Gila River confluence) and communities in highly anthropogenically altered habitats in the Salt and Gila Rivers were clustered into a single biogeographic unit (red cluster in Figure 3). Several communities in Grand Canyon tributaries (Bright Angel, Clear, Deer, Royal Arch, Shinumo, and Tapeats Creeks) clustered more closely with communities in the Mogollon Rim than with other Grand Canyon headwater tributaries (purple cluster in Figure 3).

### 3.3 Distance Decay

**FIGURE 4:**
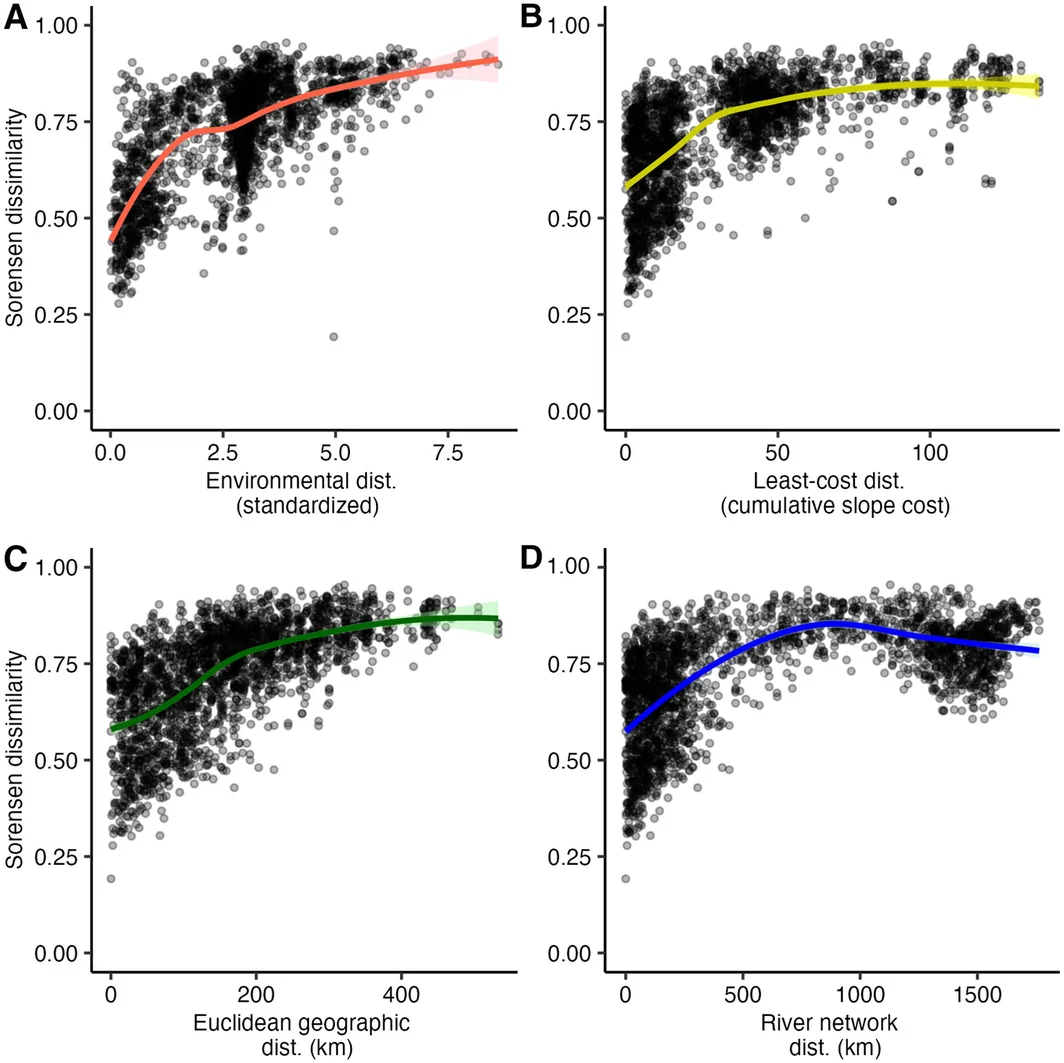
Distance decay relationships of pairwise aquatic invertebrate community dissimilarity across multiple pairwise distance metrics calculated from habitat characteristics. Points represent pairwise comparisons between sampled sites, and contour lines represent kernel density of comparisons. The four relationships were independently quantified using univariate generalized dissimilarity models (GDM) comparing community dissimilarity to the specified distance metric. (A) Community dissimilarity across pairwise environmental distances, calculated as the Euclidean distance between axes scores for a principal component analysis on scaled environmental variables. (B) Community dissimilarity across pairwise topographic least‐cost distances, measured as the calculated using a slope‐derived cost surface to represent topographic complexity in the terrestrial landscape. (C) Community dissimilarity across pairwise Euclidean geographic distances, measured as the geodesic distance between sites using Lambert conformal conic projection. (D) Community dissimilarity across pairwise river network distances, measured as the distance between sites following one‐dimensional river network connectivity of the river network. Together, these distance decay relationships highlight the dynamic influence of multiple metacommunity dynamics at local and regional scales.

Dissimilarity in community composition exhibited a positive and significant relationship with all metrics of habitat distance across the LCRB (Figure 4). A univariate GDM comparing pairwise environmental distance to community dissimilarity displayed the strongest explanatory power, explaining 50.1% of variance in observed dissimilarity that is explained by the model (I‐spline sum = 1.353, *p* ≤ 0.0001; Figure 4A, Figure S6a). A univariate GDM with topographic least‐cost distance had the second strongest explanatory power (42.6% variance explained, I‐spline sum = 1.114, *p* ≤ 0.0001; Figure 4B, Figure S6b), followed by univariate GDMs with Euclidean geographic distance (40.4% variance explained, I‐spline sum = 1.140, *p* ≤ 0.0001; Figure 4C, Figure S6c) and river network distance (38.8% variance explained, I‐spline sum = 0.804, *p* ≤ 0.0001; Figure 4D, Figure S6d).

A full GDM model, incorporating topographic, river network, and environmental distances, explained 59.7% of variance between predicted and observed dissimilarity. As with the univariate models, environmental distance had the greatest effect on community composition (I‐spline sum = 0.861, *p* ≤ 0.001), followed by topographic distance (I‐spline sum = 0.820, *p* = 0.026). River network distance, however, showed a low effect on community composition in the full model (I‐spline sum = 0.192) and did not significantly contribute to the explanatory power of the model (*p* = 0.314).

Qualitatively, the slopes of the LOESS regressions between each distance metric and community dissimilarity (Figure 4) most closely matched the HWM (Figure 1D) and the DVM (Figure 1B) models of aquatic ecological connectivity. The relationship between Sorensen dissimilarity and both Euclidean geographic and environmental distances showed a generally positive trend. With increasing river network distances, however, dissimilarity rose at low to moderate distances but declined at the furthest extents (e.g., Grand Canyon tributaries–Mogollon Rim headwater comparisons), as predicted by the HWM. This indicates that geographically sorted environmental variability is likely influential in structuring aquatic communities in the LCRB. When distances are short and environmental differences are small, pairwise comparisons showed intermediate to high Sorensen dissimilarity, consistent with the predictions of neutral community drift in the DVM.

### 3.4 Variance Partitioning

**FIGURE 5:**
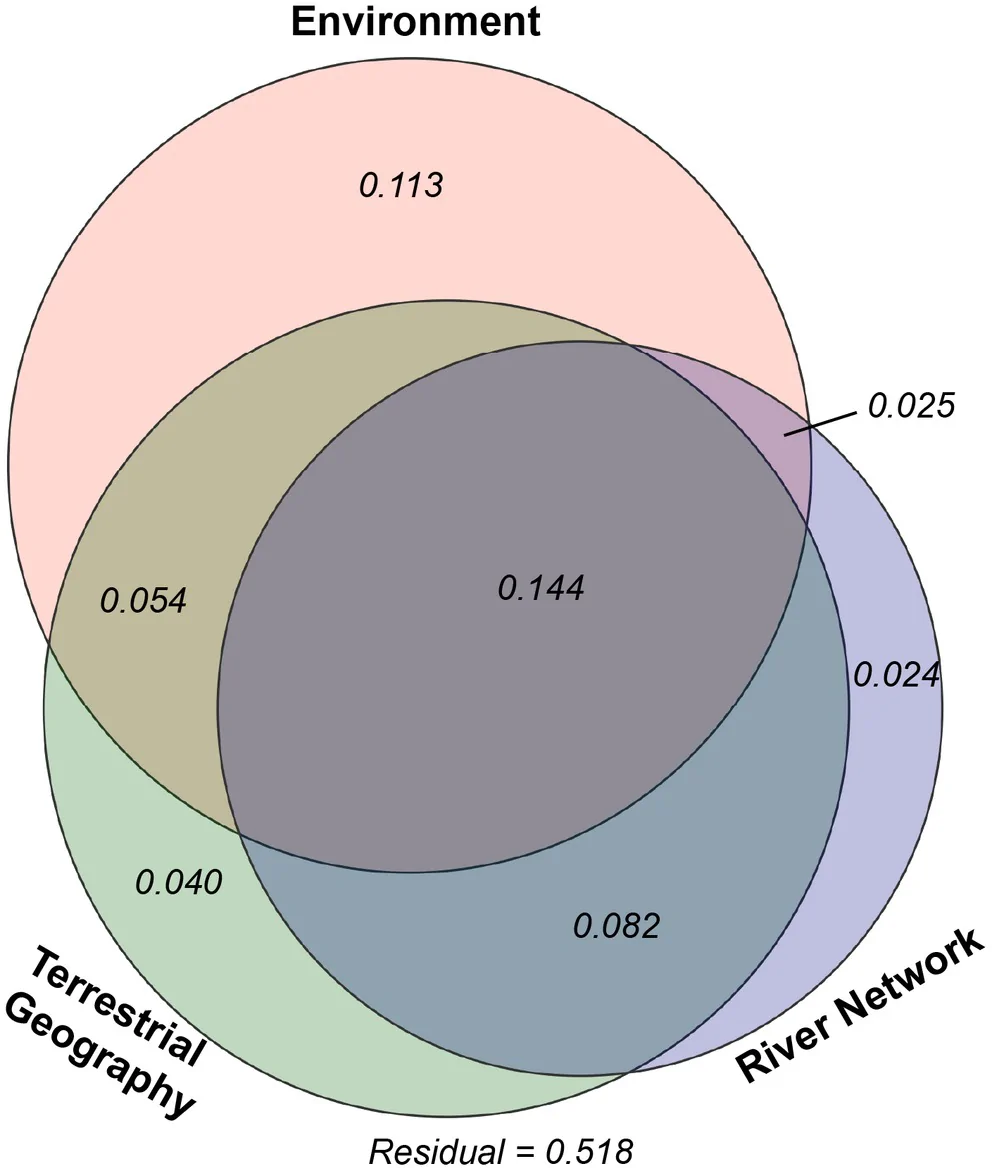
Scaled Venn diagram of variance partitioning of the influence of the environment, terrestrial geography, and the river network on aquatic invertebrate community structure in the lower Colorado River Basin. The multi‐matrix dbRDA model explained 48.2% of total variation in community structure, and the adjusted *R* ^2^ for unique and joint fractions of variance is displayed within each cell of the diagram. The joint variance between all three landscape metrics had the highest individual fraction (adj. *R* ^2^ = 0.144), followed by variance uniquely attributable to environment (adj. *R* ^2^ = 0.113) and shared variance attributable to Euclidean geography and river network connectivity (adj. *R* ^2^ = 0.082).

Given the overlapping influence of Euclidean geography and river network connectivity in the GDM models, we further investigated the shared and unique influences of habitat characteristics using a multi‐matrix dbRDA with hierarchical partitioning. Overall, this model explained 47% of variation in aquatic invertebrate community structure (Figure 5). The shared variance between the environment, Euclidean geography, and river network connectivity had the highest individual fraction (adj. *R* ^2^ = 0.144), highlighting the degree to which ecologically relevant habitat characteristics covary in the LCRB. The next highest individual fraction was variance uniquely attributable to environment (adj. *R* ^2^ = 0.113), followed by shared variance attributable to Euclidean geography and river network connectivity (adj. *R* ^2^ = 0.082). The remaining 52% of community variation was attributed to unexplained variance. Hierarchical partitioning of the individual dbRDA fractions shows that all three habitat characteristics explained a significant individual portion of community composition. Environment had the highest individual importance (*I* = 0.198, *p* < 0.0001), followed by Euclidean geography (*I* = 0.152, *p* < 0.0001) and river network connectivity (*I* = 0.125, *p* < 0.0001). The significance of all three habitat characteristics indicates that despite their covariance across the landscape, each characteristic plays a distinct role in structuring aquatic invertebrate community structure in the LCRB.

## 4 Discussion

Across the large geographic scale of the lower Colorado River Basin, we used eDNA metabarcoding to quantify the influence of spatial and environmental landscape heterogeneity on aquatic invertebrate communities. Using multiple spatially explicit analytical methods, including a novel implementation of a riverine distance decay framework, we identified environmental heterogeneity and terrestrial connectivity as significant influences on communities throughout the LCRB. Hierarchical clustering of communities revealed patterns of convergent similarity in headwater tributaries spanning a major drainage divide, further highlighting the importance of terrestrial landscape structure in shaping community composition in this river network. Overall, our results demonstrate the dual influence of spatial and environmental metacommunity dynamics in LCRB aquatic invertebrate communities and the specific influence of inter‐basin terrestrial connectivity across large spatial scales.

### 4.1 LCRB Supports Diverse Aquatic Invertebrate Communities Across Drainages

Aquatic invertebrate communities in the LCRB displayed patterns of species richness and diversity related to both spatial relationships and environmental differences among habitats. Communities had varying levels of taxonomic diversity across the basin, with mean taxonomic richness, insect richness, and EPT richness varying significantly among the five regional drainage basin designations (Figure 2). We observed the highest taxonomic diversity in the Verde River and its tributaries draining the Mogollon highlands, while the lowest taxonomic richness was observed in the Colorado River. This likely reflects differences in environmental conditions among these habitat types, with the Verde River and its tributaries having relatively unaltered flow regimes and diverse environmental conditions (Baruch et al. 2021). On the other hand, the Colorado River in the LCRB has relatively homogenous conditions and extensive anthropogenic modifications (Abernethy et al. 2021; Kennedy et al. 2016), which in turn have strong negative effects on aquatic invertebrate diversity in downstream reaches (Abernethy et al. 2021).

The structure of communities across large biogeographic areas can be conceptualized by delineating distinct clusters of species distributions (Blackman et al. 2021; Costello et al. 2017; Kreft and Jetz 2010). Despite containing no information about the spatial or environmental relationships among communities, clustering analysis of aquatic communities in the LCRB nevertheless identified ecologically relevant clusters that align to major biogeographic groupings. Two‐ and five‐cluster community groupings were supported by hierarchical clustering validation algorithms. Grouping invertebrate communities into two clusters identified a distinct cluster of communities in the predominantly canyon‐bound reaches of the Colorado River, including all mainstem communities in Grand Canyon, and the Little Colorado River and Paria River in Grand Canyon (Figure S3). Aquatic habitats in the Colorado River downstream of large hydroelectric dams, including the entire Grand Canyon reach between Glen Canyon Dam and Lake Mead, are subject to intense flow modifications that greatly reduce invertebrate biodiversity and alter community composition in the Colorado River (Abernethy et al. 2021; Déry et al. 2021; Kennedy et al. 2016). Therefore, this two‐cluster solution may reflect ecological limitations in Colorado River invertebrate communities that differentiate them from the remainder of sampled communities.

Grouping communities into five clusters identified ecologically relevant biogeographic clusters that mirror regional drainage distinctions and major environmental heterogeneities (Figure 3). These clusters generally followed a priori drainage region designations (Figure S1) and comprised communities inhabiting proximal and environmentally similar locations. Groupings identified similar communities that are primarily confined to high‐order mainstem habitats (red and orange clusters in Figure 3), mid‐order habitats at an intermediate position in the network (blue cluster in Figure 3), or low‐order habitats in the headwaters of the network (green and purple clusters in Figure 3). This delineation of community groupings across the river network highlights the region‐wide biogeographic structure of aquatic invertebrates in the LCRB, although further analyses that explicitly incorporate spatial and environmental gradients would be useful in clarifying the mechanisms driving this structure, as discussed below.

### 4.2 Environmental and Spatial Variation Interact to Influence Metacommunity Dynamics

In general, region‐wide metacommunity structure reflects nonexclusive archetypes related to local processes (i.e., species sorting along environmental gradients) and regional processes (i.e., dispersal limitations and mass effects; Leibold et al. 2004, Presley and Willig 2022). Although the relative importance of these archetypes can be highly ecosystem specific (Soininen 2014), species sorting is frequently found to be highly influential in river network metacommunities (Brown and Swan 2010; Erős et al. 2017; Heino, Melo, Siqueira, et al. 2015; Tonkin et al. 2018). Our findings in LCRB aquatic invertebrate communities support this commonality, as both analytical approaches (GDM distance‐decay and dbRDA variance partitioning) identified environmental heterogeneity as the most influential landscape variable in structuring aquatic invertebrate communities. Environmental distance and community dissimilarity displayed a strong and significant relationship in distance‐decay analysis with GDMs (Figure 4), with this relationship explaining over 50% of community variance (univariate GDM) and 26% of unique variance (multivariate GDM). The most explanatory fraction of variance identified in dbRDA variance partitioning was the three‐way shared variance attributable to the environment, the river network, and the terrestrial landscape, while the second most explanatory fraction was uniquely attributable to the environment (Figure 5). Overall, more than 70% of the explained variance was attributable to environmental heterogeneity in some way, including both shared and unique variance. Taken together, the significance of environmental heterogeneity indicates a strong role of environmental filtering (i.e., species sorting) among aquatic invertebrate communities in the LCRB (Heino, Melo, Siqueira, et al. 2015). Aquatic communities in the LCRB are found in a wide range of landscapes, ranging from semi‐arid forests to arid deserts, and are frequently isolated by harsh terrestrial topography and anthropogenetic alterations (Abernethy et al. 2021; Kennedy et al. 2016). This heterogeneity, reflected in our aggregate metric of environmental distance composed of drainage area, annual precipitation, elevation, stream type, and 100‐year flood magnitude, creates distinct habitat conditions that support the assembly of diverse invertebrate communities throughout the network (Brown and Swan 2010; Heino, Melo, and Bini 2015; Schmera et al. 2018). Thus, the high influence of environmental conditions on aquatic invertebrate communities in the LCRB may reflect species sorting according to habitat conditions.

Although environmental heterogeneity was the strongest individual predictor of community structure across both analytical methods, spatial landscape heterogeneity also contributed significantly to community structure. We identified a strong influence of covarying spatial and environmental heterogeneity in the LCRB, indicating a shared and nonexclusive influence of the environment, terrestrial landscape, and river network in structuring aquatic invertebrate communities. With variance partitioning, the fraction with the greatest explanatory power was the shared variance among all three of these landscape characteristics (Figure 5). This indicates that environmental heterogeneity may itself be spatially structured, and the contributions of local and regional processes in structuring aquatic invertebrate communities cannot be fully disentangled (Heino, Soininen, et al. 2017; Schmera et al. 2018; Suzuki and Economo 2021). Even in cases in which local environmental filtering prevails, dispersal limitations influence whether individuals of each species in the regional pool are able to sort according to their environmental preferences (Suzuki and Economo 2021; Tonkin et al. 2018). Such interactions among local and regional metacommunity dynamics are particularly influential in river networks, since environmental conditions often shift in characteristic ways throughout the network (Benda et al. 2004; Jones and Schmidt 2017) and rates and modes of dispersal differ among aquatic organisms within a community (Heino, Melo, Siqueira, et al. 2015; Tonkin et al. 2018). Therefore, the influence of local and regional dynamics may differ depending on the location of a community within the river network (Henriques‐Silva et al. 2019; Schmera et al. 2018), facilitating complex interactions among metacommunities that produce highly diverse aquatic invertebrate communities in the lower Colorado River Basin.

The 52.9% of unexplained variance in our variance partitioning analysis indicates that additional unmeasured factors and stochastic variability may influence community assembly in the LCRB. Although this level of unexplained variability is common in ecological studies (Lai et al. 2022; Mokany et al. 2022), it may also represent the influence of metacommunity dynamics beyond the archetypal “Big Four” (sensu Brown et al. 2017) that are not accounted for in our analyses. These include effects from disturbances such as floods, droughts, and forest fires (Vanschoenwinkel et al. 2013), or anthropogenic alterations such as dams and diversions (Abernethy et al. 2021). Future studies incorporating an explicit investigation of these disturbance‐related dynamics throughout the LCRB could further illuminate the specific determinants of aquatic invertebrate community structure.

### 4.3 Terrestrial Connectivity Links Aquatic Communities in the LCRB

In river ecosystems, ecological connectivity is a dominant factor influencing community assembly even in instances of strong species sorting (Suzuki and Economo 2021; Tonkin et al. 2018). Different modes of connectivity, such as dendritic connectivity in river networks or overland connectivity on the terrestrial landscape, reflect different dispersal abilities and ecological requirements of species in aquatic communities (Cañedo‐Argüelles et al. 2015; Heino, Alahuhta, et al. 2017; Henriques‐Silva et al. 2019; Peterson et al. 2013). Therefore, the relative importance of terrestrial and aquatic connectivity in an aquatic metacommunity may affect the ability of individuals to seek optimal environmental conditions and respond to disturbances (Patrick et al. 2021; Tonkin et al. 2018).

We found terrestrial connectivity to be more explanatory of aquatic invertebrate community dissimilarity patterns than river network connectivity, as demonstrated by significant distance decay relationships with topographic least‐cost distance (Figure 4B) and Euclidean geographic distance (Figure 4C). Topographic least‐cost distance explained a greater proportion of community variance than Euclidean geographic distance (42.6% vs. 40.4% with univariate GDM models), indicating that the specific topography of the LCRB influences connectivity among aquatic communities. This pattern may reflect direct overland dispersal of aquatic invertebrates (Cañedo‐Argüelles et al. 2015), or may result from spatial autocorrelation of environmental characteristics along topographic features. Many aquatic habitats in the LCRB are found in steep, topographically complex canyons, and environmental conditions can be markedly different at varying elevations and topographies, particularly among headwater habitats.

River network distance displayed a strong correlation with community dissimilarity as well in univariate GDMs (Figure 4D), although this effect was greatly diminished and not statistically significant when considered alongside topographic least‐cost distance in a multivariate GDM. Furthermore, variance partitioning identified that the shared effect of the river network and terrestrial geography was much greater than either landscape structure independently (Figure 5). This indicates that the river network and terrestrial geography covary in the LCRB, and that aquatic connectivity may be less influential than terrestrial connectivity.

Qualitative classification of ecological connectivity using the shape and fit of distance decay relationships further supported the influence of terrestrial connectivity among aquatic invertebrate communities in the LCRB. The distance decay relationship between community dissimilarity and topographic distance was generally positive (Figure 4B), while the relationship with river network distance displayed a hook‐shaped pattern in which community dissimilarity decreased at the furthest extents of river network distance (Figure 4D). This pattern matches the theoretical predictions of the HWM (Figure 1D) that biological diversity is structured by terrestrial connections among drainage basins to produce an isolation‐by‐distance pattern (Finn et al. 2007; Miller et al. 2015). When applied to metacommunity archetypes, this model describes communities in terms of dispersal limitations across the terrestrial landscape, rather than as a part of a dendritic river network (Cañedo‐Argüelles et al. 2015; Heino, Soininen, et al. 2017; Tonkin et al. 2018). However, both river network and terrestrial distance decay relationships exhibited moderate to high dissimilarity values at short geographic distances, providing additional support for the DVM (Figure 1B; Meffe and Vrijenhoek 1988). This model predicts that high and variable diversity occurs due to isolation among communities, allowing local metacommunity dynamics such as species sorting and neutral processes to drive community assembly independent from spatial relationships (Driscoll and Lindenmayer 2009; Heino, Melo, and Bini 2015). Integrating these two conceptual models, our distance decay analysis indicates that aquatic invertebrate communities in the LCRB, as a whole, are influenced by terrestrial connectivity among relatively isolated habitats, likely through the combined influence of species sorting, neutral processes, and dispersal limitations (Suzuki and Economo 2021; Tonkin et al. 2018).

Our results support the HWM and DVM and mirror similar findings from population genetic studies of individual aquatic populations (Finn et al. 2007; Miller et al. 2015; Phillipsen and Lytle 2013). By applying connectivity models first described in the population genetics literature (Finn et al. 2007; Hughes et al. 2009; Meffe and Vrijenhoek 1988) to aquatic communities, our framework offers a unique perspective to consider the metacommunity dynamics influencing aquatic invertebrates. Distance decay has a long history of use in community and metacommunity ecology (Nekola and White 1999; Soininen et al. 2007; Whittaker 1960), including in the aquatic realm (Brown and Swan 2010; Cañedo‐Argüelles et al. 2015). Our approach, which was initially proposed by Tonkin et al. (2018), allows for a co‐consideration of the multiple modes of connectivity found in river networks (Peterson et al. 2013). Although this may obscure species‐specific differences in dispersal ability and environmental tolerances, this integrative approach makes it possible to identify major pathways of connectivity with management and conservation relevance.

### 4.4 Headwater Habitats Connect Communities Across Subbasin Divisions

The observed pattern of terrestrial connectivity among isolated communities is a common occurrence for aquatic invertebrates with affinity for headwater habitats (Brown and Swan 2010; Finn et al. 2011, 2007; Schmera et al. 2018). Environmental heterogeneity tends to be highest in headwater streams (Meyer et al. 2007), meaning that both spatial and environmental factors influence aquatic metacommunity structure when terrestrial connectivity is observed (Heino, Melo, and Bini 2015). Hierarchical clustering of aquatic invertebrate communities identified a group of compositionally similar communities spanning the headwaters of the Verde River and the small tributaries within Grand Canyon (purple cluster in Figure 3). This cluster of similarity does not follow subbasin structure but rather connects environmentally and geographically similar communities inhabiting high‐elevation headwater habitats, representing a biogeographic unit characterized by convergent local habitat conditions. This demonstrates the importance of terrestrial connectivity and location‐dependent environmental conditions when considering a broadscale biogeographic perspective of metacommunity dynamics in aquatic ecosystems (Heino, Soininen, et al. 2017; Schmera et al. 2018; Viana et al. 2016).

## 5 Conclusions

Throughout the lower Colorado River Basin, we identified diverse aquatic invertebrate communities structured by a combination of local and regional metacommunity dynamics. The spatial upscaling enabled by eDNA metabarcoding enabled sampling of many diverse habitats (Blackman et al. 2024), allowing for direct comparisons of aquatic habitats ranging from small, free‐flowing headwater tributaries to anthropogenically altered high‐order rivers. Quantifying aquatic invertebrate communities at this expansive spatial scale allowed us to connect metacommunity dynamics and landscape structure to investigate ecological connectivity and metacommunity structure in LCRB aquatic habitats (Ricklefs and Jenkins 2011; Taylor et al. 1993; Tonkin et al. 2018), revealing a community structure driven by the abiotic environment and the terrestrial landscape. This highlights the ways in which local and regional metacommunity dynamics interact to promote ecological similarity in disparate reaches of the river network. The structure of aquatic communities along gradients of environmental similarity and terrestrial spatial relationships illustrates that river network structure is just one aspect of landscape structure, and that relationships among communities within, across, and among networks contribute to landscape ecological patterns of biodiversity.

## Author Contributions

**Jared W. Freedman:** conceptualization (lead), formal analysis (lead), investigation (lead), methodology (lead), visualization (lead), writing – original draft (lead), writing – review and editing (lead). **Theodore A. Kennedy:** conceptualization (supporting), funding acquisition (lead), investigation (equal), methodology (equal), project administration (equal), writing – original draft (supporting), writing – review and editing (supporting). **David A. Lytle:** conceptualization (supporting), formal analysis (supporting), funding acquisition (equal), investigation (equal), methodology (equal), project administration (lead), supervision (lead), writing – original draft (supporting), writing – review and editing (supporting).

## Funding

This work was supported by US Geological Survey/National Park Service Water Quality Partnership program and the Bureau of Reclamation's Glen Canyon Dam Adaptive Management Program.

## Conflicts of Interest

The authors declare no conflicts of interest.

## Supporting information


**Figure S1:** Map of lower Colorado River Basin (LCRB) sample sites from which eDNA samples were collected in spring 2022. Points represent sampling locations were 4x1L eDNA samples were collected. Colors correspond to drainage basin identity of each site. Legend labels correspond to the following regions: Colorado: Colorado River; GC_Tribs: Grand Canyon tributary streams; Headwaters: Mogollon Rim headwater streams; Salt/Gila: Salt‐Gila Rivers: Verde: Verde River. Base map is from USGS Global Multi‐resolution Terrain Elevation Data 2010 (GMTED2010), and river shapefiles are from USGS National Hydrography Dataset 2023.
**Figure S2:** Biplot of lower Colorado River Basin sites based on Principle Component Analysis (PCA) scores obtained from local environmental characteristics. Euclidean distance between PCA scores was used to calculate a composite metric of environmental distance.
**Figure S3:** Morgan's Eigenvector Maps (MEMs) derived from Euclidean geographic distances between sampling sites in the lower Colorado River Basin (*n* = 70). *X*‐ and *y*‐axes of each panel represent geographic latitude and longitude, respectively. Each panel represents a statistically significant MEM axis, determined by forward selection. MEM axes are ordered by decreasing percentage of community variation (*R* ^2^) and are numbered according to spatial scale: broad (MEM1‐3), intermediate (MEM4‐9), and fine (MEM > 10).
**Figure S4:** Symmetrical Morgan's Eigenvector Maps (sMEMs) derived from river network distances between sampling sites in the lower Colorado River Basin (*n* = 70). *X*‐ and *y*‐axes of each panel represent geographic latitude and longitude, respectively. Each panel represents a statistically significant MEM axis, determined by forward selection. MEM axes are ordered by decreasing percentage of community variation (*R* ^2^) and are numbered according to spatial scale: broad (MEM1‐3), intermediate (MEM4‐9), and fine (MEM > 10).
**Figure S5:** Two group hierarchical clustering of aquatic invertebrate communities in the lower Colorado River Basin (LCRB). (A) Dendrogram of community similarity displaying two clusters. (B) Map of sampled communities in the LCRB, with point colors corresponding to cluster identity. This dendrogram has the same dendritic topology as Figure 3, with different cluster coloring to indicate two clusters Dashed boxes outline map insets in regions of higher site density: (C) Grand Canyon, and (D) Verde River Basin. Two clusters generally divide communities into a Colorado River group (orange) and a second group containing the remaining sites (blue). Base map is from USGS Global Multi‐resolution Terrain Elevation Data 2010 (GMTED2010), and river shapefiles are from USGS National Hydrography Dataset 2023.
**Figure S6:** I‐splines from three univariate Generalized Dissimilarity Models comparing community dissimilarity (Sorensen dissimilarity) with measures of landscape distance. The maximum height of each spline represents the relative importance of the predictor, and the shape of the curve reflects the rate of community change with increasing distance. (A) Environmental distance (env_dist; the composite distance from principal component analysis of measured environmental variables) is the strongest predictor, explaining 50.1% of community variation (I‐spline sum = 1.353). (B) Topographic least‐cost distance (lcd) is the second strongest predictor, explaining 42.6% of community variation (I‐spline sum = 1.114). (C) Euclidean geographic distance (Geographic) is the third strongest predictor, explaining 40.4% of community variation (I‐spline sum = 1.140). (D) River network distance (riv_dist) is the fourth strongest predictor, explaining 38.8% of community variation (I‐spline sum = 0.804).
**Table S1:** Lower Colorado River Basin eDNA sampling locations. All locations were sampled between March and May 2022.
**Methods S1**. eDNA Extraction Protocol (with QIAGEN DNeasy Blood and Tissue Kit, Germantown, MD, USA).
**Methods S2**. Polymerase chain reactions and cycling conditions.
**Methods S3**. Bioinformatics pipeline.

## Data Availability

Data and code used for bioinformatic and statistical analyses will be available via Dryad (DOI: https://doi.org/10.5061/dryad.573n5tbnp). The eDNA metabarcoding sequences used in this study will be available to editors and reviewers via NCBI BioProject PRJNA1347436: https://www.ncbi.nlm.nih.gov/bioproject/PRJNA1347436/.
