# Supplementary material for: The Further You Are, the Closer You Get: Environmental Similarity Connects Aquatic Invertebrate Communities Across the Terrestrial Landscape in a Large River Network
Source: Ecol Evol. 2026 Sep 21;16(9):e74302. doi: 10.1002/ece3.74302 (PMC13593366; doi:10.1002/ece3.74302)
Supplement: Supplementary file 1 [file ECE3-16-e74302-s001.docx]

**Appendix**

Environmental and spatial heterogeneities shape aquatic communities at tributary confluences in a desert river network

Jared W. Freedman, Theodore A. Kennedy, Molly K. Burke, David A. Lytle

*Ecology* *and Evolution*

Any use of trade, product, or firm names is for descriptive purposes only and does not imply endorsement by the US government.

Figure S1. Map of Lower Colorado River Basin (LCRB) sample sites from which eDNA samples were collected in spring 2022. Points represent sampling locations were 4x1L eDNA samples were collected. Colors correspond to drainage basin identity of each site. A. LCRB sampling locations. Dashed boxes outline the boundaries of the inset maps. B. Inset map of Grand Canyon sites. C. Inset map of Verde River and Mogollon Rim Headwater sites. Legend labels correspond to the following regions: Colorado: Colorado River; GC_Tribs: Grand Canyon tributary streams; Headwaters: Mogollon Rim headwater streams; Salt/Gila: Salt-Gila Rivers: Verde: Verde River.

**Figure S2.** Biplot of Lower Colorado River Basin sites based on Principle Component Analysis (PCA) scores obtained from local environmental characteristics. Euclidean distance between PCA scores was used to calculate a composite metric of environmental distance.

**Figure S3.** Morgan’s Eigenvector Maps (MEMs) derived from Euclidean geographic distances between sampling sites in the Lower Colorado River Basin (n=70). X- and y-axes of each panel represent geographic latitude and longitude, respectively. Each panel represents a significant MEM axis, determined by forward selection. MEM axes are ordered by decreasing percentage of community variation (R2), and are numbered according to spatial scale: broad (MEM1-3), intermediate (MEM4-9), and fine (MEM >10).

**Figure S4.** Symmetrical Morgan’s Eigenvector Maps (sMEMs) derived from river network distances between sampling sites in the Lower Colorado River Basin (n=70). X- and y-axes of each panel represent geographic latitude and longitude, respectively. Each panel represents a significant MEM axis, determined by forward selection. MEM axes are ordered by decreasing percentage of community variation (R2), and are numbered according to spatial scale: broad (MEM1-3), intermediate (MEM4-9), and fine (MEM >10).

Figure S5. Two group hierarchical clustering of aquatic invertebrate communities in LCRB. A. Dendrogram of community similarity displaying two clusters. B. Map of sampled communities in the Lower Colorado River Basin, with point colors corresponding to cluster identity. Dashed boxes outline map insets in regions of higher site density: C. Grand Canyon, and D. Verde River basin. Two clusters generally divide communities into a Colorado River group (orange) and a second group containing the remaining sites (blue).

**Figure S6.** I-splines from three univariate Generalized Dissimilarity Models comparing community dissimilarity (Sorensen dissimilarity) with measures of landscape distance. The maximum height of each spline represents the relative importance of the predictor, and the shape of the curve reflects the rate of community change with increasing distance. **A.** Environmental distance (env_dist; the composite distance from PCA of measured environmental variables) is the strongest predictor, explaining 50.1% of community variation (I-spline sum = 1.353). **B.** Topographic least-cost distance (lcd) is the second strongest predictor, explaining 42.6% of community variation (I-spline sum = 1.114). **C.** Euclidean geographic distance (Geographic) is the third strongest predictor, explaining 40.4% of community variation (I-spline sum = 1.140). **D.** River network distance (riv_dist) is the fourth strongest predictor, explaining 38.8% of community variation (I-spline sum = 0.804).

Table S1. Lower Colorado River Basin eDNA sampling locations. All locations were sampled between March and May 2022.

| Site | Site Name | Drainage Region | Longitude | Latitude | Elev-ation (m) |
| --- | --- | --- | --- | --- | --- |
| CR01 | Colorado River – Grand Canyon Mile 01 | Colorado River | -111.59919 | 36.86008 | 956 |
| CR19 | Colorado River – Grand Canyon Mile 19 | Colorado River | -111.76045 | 36.64823 | 914 |
| CR52 | Colorado River – Grand Canyon Mile 52 | Colorado River | -111.85820 | 36.30742 | 852 |
| CR61 | Colorado River – Grand Canyon Mile 61 | Colorado River | -111.79884 | 36.19486 | 827 |
| CR76 | Colorado River – Grand Canyon Mile 76 | Colorado River | -111.90793 | 36.04577 | 786 |
| CR84 | Colorado River – Grand Canyon Mile 84 | Colorado River | -112.03596 | 36.08198 | 749 |
| CR88 | Colorado River – Grand Canyon Mile 88 | Colorado River | -112.09086 | 36.10061 | 742 |
| CR95 | Colorado River – Grand Canyon Mile 95 | Colorado River | -112.20887 | 36.09947 | 716 |
| CR97 | Colorado River – Grand Canyon Mile 97 | Colorado River | -112.23035 | 36.11466 | 713 |
| CR98 | Colorado River – Grand Canyon Mile 98 | Colorado River | -112.24236 | 36.13465 | 708 |
| CR109 | Colorado River – Grand Canyon Mile 109 | Colorado River | -112.34861 | 36.23716 | 670 |
| CR117 | Colorado River – Grand Canyon Mile 117 | Colorado River | -112.45041 | 36.19737 | 654 |
| CR134 | Colorado River – Grand Canyon Mile 134 | Colorado River | -112.46287 | 36.33601 | 729 |
| CR136 | Colorado River – Grand Canyon Mile 136 | Colorado River | -112.50802 | 36.38857 | 597 |
| CR144 | Colorado River – Grand Canyon Mile 144 | Colorado River | -112.62899 | 36.39213 | 581 |
| CR148 | Colorado River – Grand Canyon Mile 148 | Colorado River | -112.67237 | 36.34441 | 562 |
| CR157 | Colorado River – Grand Canyon Mile 157 | Colorado River | -112.76188 | 36.30909 | 570 |
| CR175 | Colorado River – Grand Canyon Mile 175 | Colorado River | -113.01760 | 36.23796 | 519 |
| CR180 | Colorado River – Grand Canyon Mile 180 | Colorado River | -113.08646 | 36.19615 | 508 |
| CR205 | Colorado River – Grand Canyon Mile 205 | Colorado River | -113.35014 | 36.01936 | 457 |
| CR215 | Colorado River – Grand Canyon Mile 215 | Colorado River | -113.30880 | 35.88674 | 429 |
| CR226 | Colorado River – Grand Canyon Mile 226 | Colorado River | -113.37219 | 35.76689 | 408 |
| CR229 | Colorado River – Grand Canyon Mile 229 | Colorado River | -113.42411 | 35.75110 | 394 |
| CR246 | Colorado River – Grand Canyon Mile 246 | Colorado River | -113.64673 | 35.82343 | 363 |
| CR275 | Colorado River – Grand Canyon Mile 275 | Colorado River | -113.92082 | 36.09586 | 347 |
| CRBH | Colorado River Below Hoover Dam | Colorado River | -114.73017 | 35.96346 | 198 |
| CRDD | Colorado River below Davis Dam | Colorado River | -114.62925 | 35.09205 | 149 |
| CRPD | Colorado River below Parker Dam | Colorado River | -114.21524 | 34.20787 | 112 |
| CRPV | Colorado River below Palo Verde Dam | Colorado River | -114.52601 | 33.60993 | 81 |
| CRUG | Colorado River near Gila confluence | Colorado River | -114.55765 | 32.72049 | 38 |
| BA | Bright Angel Creek | Grand Canyon Tributary | -112.09199 | 36.10031 | 742 |
| Bou | Boucher Creek | Grand Canyon Tributary | -112.23135 | 36.11537 | 713 |
| CLR | Clear Creek | Grand Canyon Tributary | -112.03574 | 36.08244 | 749 |
| Col | Columbine Fall | Grand Canyon Tributary | -113.92123 | 36.09563 | 347 |
| Cry | Crystal Creek | Grand Canyon Tributary | -112.24406 | 36.13552 | 708 |
| Deer | Deer Creek | Grand Canyon Tributary | -112.50613 | 36.39220 | 597 |
| Dia | Diamond Creek | Grand Canyon Tributary | -113.37134 | 35.76523 | 408 |
| Hav | Havasu Creek | Grand Canyon Tributary | -112.76152 | 36.30660 | 570 |
| Her | Hermit Creek | Grand Canyon Tributary | -112.21000 | 36.09880 | 716 |
| Kan | Kanab Creek | Grand Canyon Tributary | -112.63283 | 36.39234 | 581 |
| LCR | Little Colorado River | Grand Canyon Tributary | -111.79591 | 36.19034 | 827 |
| Mat | Matkatamiba Creek | Grand Canyon Tributary | -112.67444 | 36.34370 | 562 |
| Nank | Nankoweap Creek | Grand Canyon Tributary | -111.85848 | 36.30678 | 852 |
| PAR | Paria River | Grand Canyon Tributary | -111.60121 | 36.85887 | 956 |
| RAC | Royal Arch Creek | Grand Canyon Tributary | -112.45058 | 36.19695 | 654 |
| Shi | Shinumo Creek | Grand Canyon Tributary | -112.34925 | 36.23766 | 670 |
| Spe | Spencer Creek | Grand Canyon Tributary | -113.64791 | 35.82368 | 363 |
| Spr | Spring Creek | Grand Canyon Tributary | -113.35147 | 36.01869 | 457 |
| Tap | Tapeats Creek | Grand Canyon Tributary | -112.46967 | 36.37108 | 729 |
| Tra | Travertine Canyon | Grand Canyon Tributary | -113.42432 | 35.75077 | 394 |
| TS | Three Springs Creek | Grand Canyon Tributary | -113.30826 | 35.88571 | 429 |
| VW | Vulcan's Well | Grand Canyon Tributary | -113.08492 | 36.19630 | 508 |
| SCL_R | Sycamore Canyon - Lower - Riffle | Mogollon Rim | -112.06934 | 34.86939 | 1090 |
| SCU_R | Sycamore Canyon - Upper - Riffle | Mogollon Rim | -112.06453 | 34.90308 | 1139 |
| WBC_LR | Wet Beaver Creek - Lower - Riffle | Mogollon Rim | -111.67225 | 34.67494 | 1226 |
| WBC_U | Wet Beaver Creek - Upper | Mogollon Rim | -111.57610 | 34.68757 | 1537 |
| WCC_L | West Clear Creek - Lower - Riffle | Mogollon Rim | -111.66924 | 34.53278 | 1138 |
| WCC_U | West Clear Creek - Upper | Mogollon Rim | -111.47649 | 34.56086 | 1731 |
| WFOC_L | West Fork Oak Creek - Lower - Riffle | Mogollon Rim | -111.74793 | 34.98811 | 1615 |
| WFOC_U | West Fork Oak Creek - Upper | Mogollon Rim | -111.77091 | 35.00058 | 1678 |
| GRGD | Gila River at Gillespie Dam | Salt-Gila Rivers | -112.76788 | 33.22881 | 226 |
| GRY | Gila River near Colorado confluence (Yuma) | Salt-Gila Rivers | -114.55755 | 32.71941 | 38 |
| SRRS | Salt River at Rio Salado Park | Salt-Gila Rivers | -112.08131 | 33.41942 | 314 |
| SRUV | Salt River above Verde | Salt-Gila Rivers | -111.65668 | 33.54808 | 405 |
| VRBB | Verde River below Bartlett Dam | Verde River | -111.68812 | 33.95956 | 564 |
| VRBH | Verde River below Horseshoe Dam | Verde River | -111.68818 | 33.95941 | 563 |
| VRHW | Verde River headwaters | Verde River | -112.40225 | 34.86755 | 1285 |
| VROC | Verde River above Oak Creek | Verde River | -111.94277 | 34.67883 | 969 |
| VRSC | Verde River above Sycamore | Verde River | -112.07873 | 34.86314 | 1082 |
| VRWC | Verde River above West Clear Creek | Verde River | -111.83478 | 34.50528 | 914 |

**Supplemental Methods S1.** eDNA Extraction Protocol (with Qiagen DNeasy Blood and Tissue Kit)

1. Spray down workspace with 1.5% active sodium hypochloride solution (20% Clorox bleach), let bleach spray sit for 5 minutes, and wipe down. Spray down workspace with 70% ethanol and wipe down immediately
2. Add 15µL proteinase K to samples (for filter in ~1.5mL ATL buffer)
3. Incubate collection tubes at 56°C for at least 3 hours, with occasional vortexing when possible
4. Add 200uL of AE buffer into a 1.5mL tube and put on heat rack at 56°C
5. Vortex samples for 15 seconds
6. Transfer all liquid to new 1.5mL low-adhesion tubes in 700µL aliquots (3 tubes for a 2mL collection tube)
7. Add 600µL of AL buffer and 600µL of 100% EtOH to each aliquot
8. Vortex vigorously
9. Pipet the mixture sequentially (650uL max) into a DNeasy Mini spin column and centrifuge at 10,000rpm for 1 min
10. Discard flowthrough and repeat step 8 until all of the sample has been filtered
11. Place DNeasy Mini spin column in a new collection tube, add 500uL of Buffer AW1, and centrifuge for 1min at 10,000 rpm. Discard flowthrough and collection tube.
12. Place DNeasy Mini spin column in a new collection tube, add 500uL of Buffer AW2, and centrifuge for 3 min at 10,000 rpm. Discard flowthrough.
13. Spin empty spin column for 1min at 10,000 rpm to make sure all buffer has flowed through.
14. Transfer spin column to a new 1.5mL low-adhesion tube
15. Add 100uL of warm AE buffer, then transfer to room temp rack and incubate at RT for 10 minutes.
16. Centrifuge for 1 min at 10,000 rpm.
17. Re-elute DNA in the same spin column, incubate for 10 min at RT, and centrifuge for 1 minute at 10,000 rpm.
18. Store DNA at -20°C

**Supplemental Methods S2.** PCR Reactions and Cycling Conditions

Reaction and cycling conditions were adapted from Supplemental Information S1 in Leese et al. (2021).

First PCR (target amplification):

1^st^ PCR reactions contained 3µL template DNA, 1X Multiplex PCR Master Mix (Qiagen Multiplex PCR Plus kit), 0.5X CoralLoad dye (Qiagen), 0.1µM of each primer (fwhF2/EPDTr2n), and H_2_O to 25µL.

1^st^ PCR cycling conditions consisted of 5 min initial denaturation at 95°C, followed by a touchdown of 10 cycles of 30 s at 95 °C, 90 s at 64 °C (- 1 °C each cycle) and 30 s at 72 °C and additional 25 cycles of 30 s at 95 °C, 90 s at 54 °C and 30 s at 72 °C, followed by a final elongation for 10 min at 68 °C.

Second PCR (barcoding):

2^nd^ PCR reaction contained 1µL PCR product, 1X Multiplex PCR Master Mix (Qiagen Multiplex PCR Plus kit), 0.5X CoralLoad dye (Qiagen), 0.1µM of each unique indexing primer, and H_2_O to 25µL.

2^nd^ PCR cycling conditions consisted of 5 min initial denaturation at 95 °C followed by 18 cycles of 30 s at 95°C and 2 min at 72 °C, followed by a final elongation for 10 min at 68 °C.

**Supplemental Methods S3.** Bioinformatics Pipeline

Demultiplexed Illumina reads were processed, filtered, and clustered using the R package JAMP V0.77 (Elbrecht et al. 2018) unless otherwise stated:

1) Pair ended reads were merged using Merge_PE(), and non-paired reads were discarded.
2) Primer sequences were trimmed from paired reads using Cutadapt().
3) Reads were filtered for sequences of expected amplicon length (142+/-10bp) using Minmax() and for sequences with expected error greater than 0.5 with Max_ee(). Sequences that did not pass these filters were discarded.
4) Remaining reads were denoised with an alpha of 5 using Denoise().
5) Reads were clustered into OTUs using a 3% similarity threshold.
6) OTUs representing nuclear mitochondrial pseudogenes (NUMTs) and other erroneous sequence motifs were removed using metaMATE v0.4.3 (Andújar et al. 2021).

**Appendix References**

Andújar, C., Creedy, T.J., Arribas, P., López, H., Salces-Castellano, A., Pérez-Delgado, A.J., Vogler, A.P., Emerson, B.C., 2021. Validated Removal of Nuclear Pseudogenes and Sequencing Artifacts From Mitochondrial Metabarcoding Data. *Molecular Ecology Resources* 21, 1772–1787. <https://doi.org/10.1111/1755-0998.13337>

Elbrecht, V., Vamos, E.E., Steinke, D., Leese, F., 2018. Estimating Intraspecific Genetic Diversity From Community DNA Metabarcoding Data. *PeerJ* 6, e4644. <https://doi.org/10.7717/peerj.4644>

Leese, F., Sander, M., Buchner, D., Elbrecht, V., Haase, P., Zizka, V.M.A., 2021. Improved Freshwater Macroinvertebrate Detection From Environmental DNA Through Minimized Nontarget Amplification. *Environmental DNA* 3, 261–276. <https://doi.org/10.1002/edn3.177>
